# Impact of Aldosterone on the Failing Myocardium: Insights from Mitochondria and Adrenergic Receptors Signaling and Function

**DOI:** 10.3390/cells10061552

**Published:** 2021-06-19

**Authors:** Mariona Guitart-Mampel, Pedro Urquiza, Jordana I. Borges, Anastasios Lymperopoulos, Maria E. Solesio

**Affiliations:** 1 Department of Biology, College of Arts and Sciences, Rutgers University, Camden, NJ 08103, USA; mg1616@camden.rutgers.edu (M.G.-M.); pu23@camden.rutgers.edu (P.U.); 2 Department of Pharmaceutical Sciences, College of Pharmacy, Nova Southeastern University, Fort Lauderdale, FL 33328, USA; jb3837@mynsu.nova.edu

**Keywords:** adverse remodeling, aldosterone, signaling crosstalk, G protein-coupled receptor (GPCR), heart failure, mineralocorticoid receptor, mitochondria, mitochondrial dysfunction, mitochondrial dynamics, mitochondrial bioenergetics

## Abstract

The mineralocorticoid aldosterone regulates electrolyte and blood volume homeostasis, but it also adversely modulates the structure and function of the chronically failing heart, through its elevated production in chronic human post-myocardial infarction (MI) heart failure (HF). By activating the mineralocorticoid receptor (MR), a ligand-regulated transcription factor, aldosterone promotes inflammation and fibrosis of the heart, while increasing oxidative stress, ultimately induding mitochondrial dysfunction in the failing myocardium. To reduce morbidity and mortality in advanced stage HF, MR antagonist drugs, such as spironolactone and eplerenone, are used. In addition to the MR, aldosterone can bind and stimulate other receptors, such as the plasma membrane-residing G protein-coupled estrogen receptor (GPER), further complicating it signaling properties in the myocardium. Given the salient role that adrenergic receptor (ARs)—particularly βARs—play in cardiac physiology and pathology, unsurprisingly, that part of the impact of aldosterone on the failing heart is mediated by its effects on the signaling and function of these receptors. Aldosterone can significantly precipitate the well-documented derangement of cardiac AR signaling and impairment of AR function, critically underlying chronic human HF. One of the main consequences of HF in mammalian models at the cellular level is the presence of mitochondrial dysfunction. As such, preventing mitochondrial dysfunction could be a valid pharmacological target in this condition. This review summarizes the current experimental evidence for this aldosterone/AR crosstalk in both the healthy and failing heart, and the impact of mitochondrial dysfunction in HF. Recent findings from signaling studies focusing on MR and AR crosstalk via non-conventional signaling of molecules that normally terminate the signaling of ARs in the heart, i.e., the G protein-coupled receptor-kinases (GRKs), are also highlighted.

## 1. Introduction

Heart failure (HF) is a major health concern worldwide, affecting 10% of the adult population [1]. A complex syndrome, it is defined by damaged contractile performance of the myocardium, leading the heart to undersupply blood to the peripheral tissues [1]. The mineralocorticoid aldosterone plays a key role in this pathology [2]. Specifically, aldosterone regulates electrolyte homeostasis and blood pressure and volume. It also modulates cardiac adverse remodeling post-myocardial infarction (MI) via direct effects on the myocardium. Therefore, aldosterone antagonists, such as spironolactone and eplerenone, reduce morbidity and mortality in human HF and are part of the cornerstone pharmacotherapy of advanced stage disease [3]. Aldosterone has multiple effects in cardiac myocytes, fibroblasts, coronary endothelial cells, and infiltrating immune cells (e.g., macrophages), including its direct involvement in the dysregulation of cardiac adrenergic receptors (ARs), which mediate the effects of norepinephrine (NE) and epinephrine (Epi) on the heart. The ARs comprise nine different subtypes in mammals: α1A, α1B, α1D, α2A, α2B, α2C, β1, β2, and β3. All nine are class-A G protein-coupled receptors (GPCRs), and most play pivotal roles in regulating cardiac function, physiology, and pathophysiology [2]. Moreover, a major molecular hallmark of human chronic HF is the dysfunction/dysregulation of βAR signaling, leading to diminished inotropic and adrenergic reserves of the failing heart. Consequently, the myocardium no longer properly responds to NE or Epi, i.e., by increasing its contractile function [2,4,5,6]. This marks the “point of no return” for the heart, upon which cardiac function no longer operates on the Frank–Starling curve of cardiac elasticity; instead, it operates under the Laplace’s law of increased filling pressure, proportionally increasing the free ventricular wall pressure, ultimately causing cardiac hypertrophy, dilatation, and other remodeling processes culminating in reduced ejection fraction and cardiac output, i.e., in systolic HF [2].

At the cellular level, mitochondrial dysfunction has been described as a crucial component of the etiopathology of multiple diseases [7,8,9,10,11,12,13,14], including cardiac dysfunction induced by elevated aldosterone [15]. Moreover, it is known that aldosterone increases the production of reactive oxygen species (ROS), via NADPH oxidase [16,17]. Consequently, increased oxidative stress, cardiac mitochondrial dysfunction, and accelerated cardiac aging have been described in different mammalian models of HF [18,19,20]. Specifically, this increased ROS production induces in the organelle a loss of metabolic capacity, including dysregulated dynamics and bioenergetics. At the molecular level, the effects of aldosterone in mitochondria are mediated by the down regulator of the A-kinase anchor protein (AKAP)-12, a protein which has a mineralocorticoid receptor (MR) [21]. Furthermore, another crucial contributor towards the progression of systolic HF is the impaired production of high energy phosphates [18].

Since both mitochondrial and AR dysfunction in the heart are pathological hallmarks of the detrimental actions of aldosterone in HF, the present review focuses on the impact of aldosterone on these two key parameters regulating cardiac function. We first summarize the current experimental evidence for the cardiac mitochondrial dysfunction underlying HF, followed by an overview of the cardiac AR dysregulation underlying chronic HF. We then present the evidence on the role that aldosterone plays in the impairment of both mitochondrial and AR signaling and function in the myocardium, leading to, and/or accelerating, the development of chronic HF. Also highlighted are some of the latest findings on the crosstalk between aldosterone, mitochondria, and cardiac AR signaling with novel therapeutic repercussions for human HF treatment.

## 2. Aldosterone and Mitochondrial Dysfunction in the Failing Heart

A set of highly complex and interconnected cellular and molecular mechanisms underlies HF [22,23], some of these mechanisms are closely related to mitochondria, as in the case of biogenesis and turnover of the organelle, and ATP production [23]. Moreover, the preservation of mitochondrial physiology is crucial for cell survival, especially in non-dividing cells with high demands of energy, such as cardiomyocytes [24]. In these cells, mitochondrial dysfunction has been linked to increased apoptosis, ultimately leading to cardiomyopathy, one of the major causes of HF [24,25]. The explanation for this may be that almost 90% of the energy demands of the heart are met by the mitochondrial oxidative phosphorylation (OXPHOS), which is the major source of ATP in mammals [26]. ATP synthesis is paired with cell respiration and oxygen consumption in the electron transport chain (ETC), composed of four complexes (I, II, III and IV), that ATP synthase, and two mobile electron carriers (coenzyme Q and cytochrome C) [27]. As a result of OXPHOS, mitochondria are also the leading source of ROS generation within mammalian cells. In these cells, ROS are the main contributors to increased oxidative stress, under pathological conditions [28]. Interestingly, ROS are closely related to high energy phosphates in mitochondrial, as they are both produced during OXPHOS. One important source of high energy phosphate inside mitochondria is inorganic polyphosphate (polyP) [29,30,31,31]. In fact, the involvement of polyP on heart physiology, has been already demonstrated in cardiac myocytes [32,33,34].

Some studies have shown the deleterious effects of a high-fat diet (HFD) widely associated with HF, on mitochondrial function in the myocardium of male rats [35]. These rats showed abnormal expression of genes and proteins involved on mitochondrial dynamics; decreased number of copies of mitochondrial DNA; reduced enzymatic activities of ETC complexes I, II and III and citrate synthase—the key enzyme in the Krebs cycle; as well as decreased mitochondrial respiration and ATP levels. These data were corroborated by another study, conducted in a rabbit model of HF, using proteomics techniques, the authors showed altered levels of the main proteins involved in the cellular energy metabolism, including pyruvate dehydrogenase and mitochondrial ATP synthase [36]. Furthermore, in diabetic murine hearts (type 2), decreased levels of inner mitochondrial membrane proteins involved in bioenergetics processes, ATP synthesis, and mitochondrial protein import system; were found [37]. Type 2 diabetes is associated with reduced lifespan in humans, due to increased prevalence of HF [37]. Additionally, using left ventricular tissue obtained from HF patients undergoing heart transplantation, reduced mitochondrial oxygen respiration and reduced activity of ETC complexes and of citrate synthase were reported [38].

In another research article, upregulation of certain genes in HF patients was highlighted. Specifically, the authors found that the genes related to mitochondrial ATP synthase were those most affected. Downregulation of genes related to autophagy was also present in the same samples [39]. Finally, in another study, the presence of mitochondrial enzymatic defects in left ventricular tissue obtained from human HF patients was examined. The authors concluded that, compared with non-failing donor hearts, the activity rates of complexes I and IV, as well as of Krebs cycle enzymes such as malate dehydrogenase, were decreased in HF patients [40].

As mentioned above, the deleterious presence of increased oxidative stress, a classical consequence of dysregulated OXPHOS, has already been broadly demonstrated in the etiopathology of cardiac remodeling, the main process responsible for the development and progression of HF [25]. Corroborating these findings, different authors showed that activation of the mitochondrial detoxifying systems, which are involved in preventing and/or decreasing oxidative stress, is a valid strategy to prevent cardiac degeneration in HF [41,42,43,44,45,46]. Diverse preclinical and clinical studies have shown the protective effects of several mitochondrial-targeted molecules against HF [47,48,49,50,51]. Similarly, other authors analyzed the effects of MitoQ, a mitochondrial-addressed antioxidant [9], on the development and progression of HF induced by pressure overload. This study concluded that MitoQ restored mitochondrial membrane potential, crucial for the proper functioning and OXPHOS, and thus, of mitochondrial respiration [42].

Mitochondrial dynamics (fission and fusion) and mitophagy, all processes closely related to bioenergetics, have been also proved to be dysfunctional in HF [52]. Mitochondrial fission and fusion are in a tight equilibrium, and they are essential contributors towards maintaining healthy mitochondria by efficiently mixing mitochondrial components to compensate for any defects present in mitochondrial DNA (fusion), and by increasing the number of new mitochondria when energy demand is also increased (fission process) [53,54]. Fission also allows damaged regions of mitochondrion to be removed by mitophagy. While fission events are mediated by dynamin-related protein 1 (Drp1), which is recruited to mitochondria by MFF (Mitochondrial Fission Factor) and promotes fission thanks to its GTPase activity; mitofusin 1 and 2 (Mfn1 and Mfn2), two proteins located on the outer mitochondrial membrane, and optic atrophy 1 protein (OPA1), located in the inner mitochondrial membrane, are governing the fusion process [54]. As stated above, mitophagy, a mitochondrial-specific type of autophagy, is activated when defective mitochondria are present in cells, usually after dysfunctional fission. In this process, damaged mitochondria are surrounded by double membrane vesicles that fuse with lysosomes to eliminate the organelles [54,55].

Some studies have described dysfunctional dynamics and mitophagy in HF. For example, in a mouse model in which transverse aortic constriction (TAC) was performed to induce HF, increased mitophagy was observed around 3–7 days after TAC, as well as increasing mitochondrial translocation of Drp1. However, mitophagy was downregulated, followed by mitochondrial dysfunction [56]. Another study, also conducted using mice, showed that unbalanced OPA1 processing and mitochondrial fragmentation, often indicate signs of increased fission of the organelle, are critical in the etiopathology of HF [57]. In this work, the authors generated conditional mouse models for YmeL1l and OMA1 (two peptidases needed for appropriated OPA1 processing) finding that OPA1 proteolysis was accelerated, triggering increased mitochondrial fragmentation and altered cardiac metabolism. The authors concluded that appropriated adult myocardial function depends on balanced mitochondrial dynamics [57]. All of these studies show that proper mitochondrial physiology is key for adequate cardiac function, and that the impairment of mitochondrial physiology is considered pivotal in the progression of HF. Interestingly, this impairment could be at least partially mediated by aldosterone and MR. As mentioned above, the hormone aldosterone is one of the main volume-regulatory effectors. Consequently, it is vital for fluid and hemodynamic homeostasis in mammals, and it is a major regulator of blood pressure and heart rate in humans [43].

In the heart, one of the main effects of the binding of aldosterone to MR is the modulation of cardiac physiology through the interaction of the aldosterone–MR complex with the epidermal growth factor receptor (EGFR). Several studies show that the effect of aldosterone stimulates EGFR activation through its biding to MR, which ultimately regulates the renin/angiotensin/aldosterone system (RAAS) [46]. RAAS main physiological functions are to elevate blood volume and arterial tone by increasing sodium and water reabsorption and vascular tone [58,59,60]. Accordingly, dysregulated activation of RAAS can lead to the development of hypertension and chronically alter the blood volume, such as in renal artery stenosis [43,58,61].

Intracellularly, the stimulation of EGFR increases the production of ROS, via reduced NADPH oxidase and mitochondria [46,62]. Specifically, EGFR activates the NADPH oxidase (NOX), increasing the generation of the superoxide anion (O_2_^−^). O_2_^−^ is then converted into hydrogen peroxide (H_2_O_2_) by the superoxide dismutase enzyme (SOD). The subsequent production of H_2_O_2_ induces the opening of the mitochondrial ATP-dependent potassium channels (mitoKATP), yielding to enhanced mitochondrial O_2_^−^ production by the ETC, on a deleterious cycle [59,63]. Intriguingly, O_2_^−^, H_2_O_2_ and other mitochondrial ROS have been described as potent signaling molecules [46]. In fact, a study conducted in obese mice models, showed that the activation of MRs increases cellular senescence and mitochondrial dysfunction in the adipocyte tissue, which consequently increases vascular contractility, leading to vascular dysfunction [64]. The study showed that the aldosterone-induced oxidative stress could be mediated by AKAPs. Indeed, AKAPs play a role in the modulation of ROS synthesis by their ability to bind to the regulatory subunit of cAMP-dependent protein kinase A (PKA) [65]. Specifically, aldosterone downregulates the expression of AKAP-1 in adult human cardiac fibroblasts, which leads to increased ROS, mitochondrial dysfunction, and ultimately apoptotic cell death [21,66]. Furthermore, the aldosterone–MR signaling pathway has also shown to affect mitochondrial function through the regulation of the adenylyl-cyclase, the enzyme that synthesizes cAMP [67,68]. cAMP activates PKA, which phosphorylates Ca^2+^ channels [65,69,70]. Consequently, both increased ROS release into the cytosol and phosphorylation of these channels, including the Mitochondrial Calcium Uniporter (MCU) channel ultimately turn into the enhancement of the intracellular calcium influx into the mitochondria, with deleterious effects for these organelles, and ultimately for the whole cell [71].

Based on the above bibliography, targeting mitochondria is a promising approach in HF. In fact, some hormones such as cortisol or aldosterone, which are the physiological pathway by which MRs typically are activated and/or modulated, can be pharmacologically modulated. Moreover, AR blockage could be another plausible pharmacological strategy against HF. There are pre-clinical studies in animals using (i) Propranolol, the study showed decreased respiratory control index in cardiac mitochondria [72]; (ii) Metoprolol, the study showed inhibition of the metabolism of fatty acids into the mitochondria β oxidation, which optimizes cardiac bioenergetics, contributing to decreased decompensatory effects in HF [73]; (iii) Carvedilol, the study showed inhibition of the mitochondrial permeability transition pore opening, which decreases cell damage and increases mitochondria biogenesis [26]; and (iv) Atenolol, the study showed reduced mitochondria oxygen consumption and, consequently, ROS production [74]. However, the two only published clinical trials, which are randomized comparisons between the effects of MR blockers and placebo in patients with HF, reported no conclusive results [75,76]. Therefore, further research should be conducted in this field. Moreover, mitochondrial dysfunction in HF is closely interconnected with the effects induced by the failing hear in the rest of the cell. Therefore, understanding the extra-mitochondrial components of the etiopathology of HF, including the adrenergic system and the role of aldosterone, are crucial to search for new pharmacological targets in HF, both mitochondrial and non-mitochondrial.

## 3. Cardiac Adrenergic Receptor Signaling and Dysregulation in HF

The sympathetic nervous system (SNS) is a central actor in cardiovascular regulation. In fact, the heart receives dense noradrenergic innervation emanating from cervical and thoracic ganglia [77]. β-ARs belong to the G protein-coupled receptor (GPCR) superfamily, mediating many of the actions of norepinephrine (NE) on the heart, which is released from SNS neuronal store vesicles inside presynaptic neurons, and of epinephrine (Epi) activating these receptors via the blood circulation stemming from the adrenal medulla, where it is primarily synthesized and secreted [78]. Cardiomyocytes express various AR subtypes with the β_1_AR being the most predominant subtype under physiological conditions. Additionally, about 15% of the cardiac AR complement is the β_2_AR subtype, and the rest is comprised of β_3_AR, which has negative inotropic properties, and α_1_ARs [79,80,81] β_1_ARs and β_2_ARs promote the classic effects of SNS on the heart, i.e., positive inotropy, chronotropy, lusitropy, and dromotropy via the stimulation of the stimulatory G protein alpha subunit (G_sα_), which directly activates membrane-bound adenylyl cyclase (AC) to convert adenosine trisphosphate (ATP) to cyclic 3′,5′-adenosine monophosphate (cAMP). cAMP is a major second messenger activating PKA, a protein which phosphorylates a variety of target proteins involved in calcium handling (excitation-contraction coupling) and in contractility regulation, such as L-type Ca^2+^ channels (LTCC’s), sarcoplasmic reticulum (SR) Ca^2+^ release channels (ryanodine receptors), SR-residing Ca^2+^-ATPase (SERCA) activation via phospholamban phosphorylation, troponin-I, phospholemman, etc. [2,82]. Moreover, β_2_ARs also couples to pertussis toxin-sensitive inhibitory/other G proteins (G_i/o_), resulting in negative inotropy but increased survival (anti-apoptosis) [83,84,85]. It is important to note that in cardiac cells that are not myocytes, i.e., fibroblasts, endothelial cells, immune cells, the predominant βAR subtype is the β_2_AR, not the β_1_AR [86].

In chronic HF with reduced ejection fraction, the chronically elevated SNS activity results in adverse cardiac remodeling and diminished inotropic and adrenergic reserves [86]. Increased SNS activity portends poor prognosis in HF patients [87,88,89]. Moreover, in animal models, chronic β-agonist administration leads to heart damage and remodeling marked by cardiomyocyte loss, leukocyte infiltration, interstitial fibrosis and dysfunction [90,91]. Of note, sympathomimetics such as dobutamine and PDE3 inhibitors (e.g., milrinone) have also failed in clinical trials of chronic HF due to increased cardiac apoptosis and oxygen/metabolic demand that the failing heart fails to meet [3]. Furthermore, studies conducted in transgenic mice overexpressing cardiac βARs or downstream signaling molecules such as AC and PKA, also support the notion of enhanced cardiac toxicity exerted by the hyperactive SNS [92,93,94]. This has formed the basis for the initially extremely controversial clinical use of β-blockers in chronic HF patients. However, recent findings show that β-blockers, especially carvedilol, metoprolol, and bisoprolol, improve long-term prognosis in HF patients by counteracting the chronic toxicity of elevated catecholamines [95,96].

Like most GPCRs, β_1_ARs and β_2_ARs undergo phosphorylation by GPCR-kinases (GRKs), followed by the switching of their coupling from G proteins to β-arrestins. The latter consist of two isoforms, β-arrestin1 and -2, both of which are scaffold signaling proteins, thereby promoting non-G protein-dependent signaling [97]. There is evidence that aberrant βAR signaling is a culprit for the molecular underpinnings of HF. In fact, transgenic mice overexpressing β_2_AR fail to develop HF if crossed with mice expressing a dominant-negative mutant p38 mitogen-activated protein kinase (MAPK) in their hearts, indicating that β_2_AR signaling through p38 MAPK leads to HF [98]. In contrast, β-arrestin (β-arrestin2 in particular) signaling might be cardio-protective and pro-contractile in the post-infarct heart progressing to HF [99,100,101,102,103,104].

Interestingly, SNS hyperactivity leads to β_1_AR downregulation (i.e., total functional receptor number reduced) in human failing hearts, accompanied by β_2_AR increased desensitization, i.e., severe G-protein decoupling [105,106]. Indeed, elevated NE plasma levels correlate with significant loss of myocardial βAR function/signaling in HF patients [107,108]. β_1_AR downregulation occurs either via reduced receptor synthesis or post-translational modifications, as a result of agonist-induced desensitization which leads to β-arrestin- and clathrin-dependent receptor internalization and, ultimately, lysosomal degradation (downregulation). Alternatively, the receptor can recycle back to the plasma membrane ready to signal again upon subsequent agonist stimulation (resensitization) [2,106]. Whereas cardiac β_1_AR density is reduced in HF, β_2_AR density remains the same, which means that the β_1_AR:β_2_AR ratio becomes almost 50:50 in the failing human heart. Nevertheless, compartmentalization of β_2_AR signaling is fundamentally altered in HF, which renders this subtype equally incapable of signaling and functioning properly, like its β_1_AR counterpart [2,109]. In addition, enhanced β_2_AR signaling through G_i_ (rather than G_s_) proteins further contributes to the reduced inotropic reserve of the failing heart, which is almost exclusively dependent on cAMP [110]. Interestingly, under physiological conditions, β_2_AR is cardio-protective via G_i_ protein/phosphoinositide-3′-kinase (PI3K) activation [111]. The most abundant GRK in the heart, GRK2, is further upregulated and opposes pro-contractile signaling of both β_1_AR and β_2_AR [112,113,114,115]. Moreover, β_2_AR signaling is highly compartmentalized inside cardiomyocytes [116,117], and this subcellular targeting/anchoring of this receptor’s signaling is lost in HF [116,118]. Indeed, β_2_AR cAMP-mediated signaling is diffuse throughout the cardiac myocyte in failing hearts [119,120].

Studies in transgenic mice have revealed that β_2_AR cardiac-specific overexpression leads to age-dependent onset of HF, fibrosis, ventricular arrhythmias, and premature death [116,117,121]. Moreover, mice overexpressing β_2_AR in their cardiomyocytes also display accelerated pressure overload-precipitated HF onset, and decreased survival with enhanced adverse remodeling of the heart [122]. Interestingly, during post-myocardial infarction (MI), these mice show preservation of cardiac function and less severe HF, indicating a beneficial role for cardiac β_2_AR post-MI [123]. Chronic administration of a high doses of βAR agonists, such as isoproterenol or epinephrine, induces significant cardiac dysfunction, and β_2_AR stimulation preferentially activating G_i_ protein-mediated contractility suppression in the apical more so than in the basal region of the myocardium [124].

Although PKA, the kinase activated by cAMP, has been exhaustively studied in the heart and assigned a plethora of βAR-elicited effects in the myocardium, the role of the other effector activated by cAMP, Epac (exchange protein directly activated by cAMP), which was initially discovered in 1998 [125], has been increasingly appreciated over the past two decades. Epac has two major isoforms, Epac1 and Epac2 [126]. The latter appears mainly expressed in neuronal tissues, while Epac1 is ubiquitously expressed and quite abundant in the heart [126]. Epac1 acts as a multi-protein complex forming scaffold molecule, pretty much like β-arrestins. It activates a variety of effectors, most importantly Ca^2+^/calmodulin-dependent protein kinase (CaMKII) and the small (monomeric) G protein Rap1 [126]. Epac1 is abundant also in cardiac mitochondria, where it mediates cardiomyocyte apoptosis via Ca^2+^ overload and increases the mitochondrial permeability [127]. In fact, Epac1 promotes mitochondrial Ca^2+^ influx via VDAC1 (voltage-dependent anion-selective channel-1) and MCU [128]. Since Ca^2+^ mitochondrial levels tightly regulate and closely related to cellular respiration and ATP biosynthesis [129], Epac1 plays an important role in regulation of energy consumption in the heart. In fact, Epac1 is also known to increase ROS generation, which ultimately leads to adverse cardiac remodeling and apoptosis [130]. Moreover, at the cellular level, cardiac Epac1 has been reported to be upregulated upon chronic catecholamine administration, upon TAC-induced pressure overload, and in end-stage HF patient-derived hearts [126]. In fact, Epac1 genetic deletion protects against cardiac hypertrophy, apoptosis, and fibrosis, in response to chronic catecholamine stimulation or pressure overload [131,132]. To further support the crucial role of this protein in heart physiology, Epac1 knockout as well as Epac1 pharmacological inhibition led to cardioprotective effects in animal models of stress-induced cardiomyopathy [133].

Interestingly, β_1_AR has also been shown in heterologous systems in vitro to interact with Giα subunits of G proteins, similarly to its β_2_AR counterpart, although this fact remains still highly controversial. For example, the β-blocker carvedilol was recently shown to induce a β_1_AR-Giα interaction that supposedly facilitates subsequent β-arrestin signaling by this receptor [134]. Carvedilol has also been reported to promote β_1_AR-Giα interaction that leads to signaling via the cardioprotective PI3K/Akt/nitric oxide (NO)/protein kinase G (PKG) pathway [135]. As previously mentioned, these findings are quite controversial, given that carvedilol is an inverse agonist for G proteins at the βARs, i.e., under physiological conditions it suppresses G-protein activation by the cardiac βARs [81]. In addition, the notion that carvedilol is a β-arrestin- “biased” ligand has been challenged by a variety of studies [136,137,138]. Therefore, they await confirmation in physiologically relevant in vitro settings, and, of course, in vivo. If proven true, then it is quite plausible that the β_1_AR interacts with Giα to induce downstream cardioprotective signaling pathways that involve β-arrestins, PI3K and cGMP/PKG. However, the notion that cardiac β_1_AR signaling can turn cardioprotective in the failing heart goes directly against the consensus that selective cardiac β_1_AR downregulation, a molecular hallmark of chronic human HF, serves as an adaptive homeostatic process employed by the failing myocardium to shield itself against the chronic catecholaminergic stress in HF, which results from the chronically elevated SNS activity that accompanies and aggravates human HF [2]. Adding more complexity to cardiac βAR signaling in HF, β_2_AR was recently reported to restrict β_1_AR signaling into specific subcellular compartments [139]. This was mediated by GRK2-dependent phosphorylation and subsequent β-arrestin1-mediated phosphodiesterase (PDE)-4 recruitment.

Cardiac fibroblasts represent ~20–30% of the total cardiac cell number under physiological conditions (these percentages can vary significantly depending on the presence of diseases and on the state of this disease) [86]. In contrast to cardiac myocytes, cardiac fibroblasts mainly express the β_2_AR subtype [140]. Whether β_2_AR promotes or inhibits cardiac the proliferation and the activation of fibroblasts is still a matter of intense investigation [141]. On one hand, some studies have shown that β_2_AR may stimulate cell proliferation and pro-inflammatory/pro-fibrotic gene expression in cardiac fibroblasts [141,142,143]. In fact, GRK2 plays a key role in myocardial fibrogenesis [144] and β_2_AR has been shown to promote interleukin (IL)-6 secretion and hypertrophy [141,145]. Moreover, β_1_/β_2_AR double knockout mice exhibited significantly less interstitial fibrosis and fibrotic mediator expression (TGFβ, CTGF, collagen-III) in response to TAC, compared to control animals [146]. Furthermore, fibroblast-restricted genetic deletion of β_2_AR led to prevention of isoproterenol-induced cardiac hypertrophy, dysfunction and fibrosis in transgenic mice [91]. On the other hand, cAMP and especially its effector Epac1, has been associated with anti-fibrotic signaling in various tissues, including in the heart, and β_2_AR was recently shown to inhibit cardiac fibrosis via cAMP in vitro, in a mechanism involving cAMP and Epac1, an effect opposed by the pro-inflammatory cytokine osteopontin and GRK2 [86,147,148].

In addition to βARs, α_1_ARs are also present in mammalian cardiomyocytes, with the α_1A_ α_1B_ subtypes accounting for ~10% of total AR number in the healthy adult myocardium [149]. α_1B_AR appears to have detrimental consequences for the failing myocardium [149,150], whereas the α_1A_AR subtype seems to be beneficial in HF. Interestingly, deletion of both subtypes leads to enhanced TAC-induced apoptosis, cardiac dilatation, and reduced survival in mice [151]. On the one hand, cardiac-specific α_1B_AR overexpression results in severe pressure overload-induced hypertrophy, fibrosis, and HF [152]. On the other hand, α_1A_AR knockout increases cardiac apoptosis/necrosis in response to NE, doxorubicin, or oxidative stress [153]. In addition, α_1A_AR activation protects the heart against pressure overload through its classic G_q/11_ protein signaling [154].

From a mechanistic point of view, α_1A_AR-dependent cardioprotection is mediated by protein kinase C (PKC), a protein that promotes glucose transporter (GLUT)-1/4 activity to enhance glucose metabolism, by ERK activation to prevent apoptosis, and via pro-angiogenic signaling [154,155]. Furthermore, cardiac α_1A_AR overexpression leads to augmented contractility without accompanying hypertrophy or fibrosis [156], and α_1A_AR activation is essential for preservation of cardiac function and survival post-TAC or upon doxorubicin challenge [157,158]. Both α_1A_AR and α_1B_AR subtypes have been shown to be expressed in failing human heart, and, interestingly enough, to resist downregulation in the failing human heart [159]. This is consistent with data showing that α_1A_AR-dependent positive inotropy being intact in hearts from HF patients [160]. The extent of positive inotropy exerted by α_1A_AR remains somewhat controversial but, at the molecular level, it is presumably mediated by increased intracellular Ca^2+^ signaling and RhoA-dependent kinase (ROCK) activation [161,162]. Transient receptor potential channel (TRPC)-6 activation and subcellular translocation may also be involved [162].

Finally, with regard to cardiac α_2_ARs, NE release of NE from cardiac SNS terminals is controlled by both presynaptic α_2A_- and α_2C_ARs [163]. In fact, genetic knockout of both results in cardiac hypertrophy and HF, due to chronically elevated NE levels. Epi levels are increased also, as a result of augmented adrenal secretion [164,165]. Interestingly, α_2_ARs act as presynaptic inhibitory auto-receptors not only in cardiac SNS terminals but also in the chromaffin cells of the adrenal medulla, wherein they prevent further Epi and NE release [166]. This process is under tonic control of adrenal GRK2 (acting in concert with β-arrestin1), given that adrenal GRK2 upregulation is a molecular hallmark of the SNS hyperactivity that accompanies chronic HF [115,167,168]. Indeed, GRK2 upregulation in pre-ganglionic sympathetic nerves innervating the adrenal medulla promotes TAC-induced SNS hyperactivity [169]. Supporting this data, some authors have published that a human polymorphic α_2B_AR variant resistant to GRK2-dependent phosphorylation and desensitization provides augmented sympatholysis in a transfected chromaffin cell line [170]. Another human α_2_AR polymorphic variant, the Ins/Del322–325 α_2C_AR, which is quite frequent in African-American HF patients and which shows diminished sympatholytic capacity to inhibit NE release from cardiac SNS terminals, significantly affects HF risk and prognosis in patients, when this is examined in conjunction with the hyperfunctional Agr389 variant of the human β_1_AR [171]. Indeed, the combined presence of Arg389 β_1_AR together with Del322–325 α_2C_AR can prognosticate the clinical response to the β-blocker bucindolol in black HF patients [172].

## 4. Aldosterone-Induced Adrenergic Receptor Dysfunction in the Failing Myocardium

Aldosterone is produced from the adrenal cortex in response to hyperkalemia or angiotensin II stimulation of its type 1 receptor (AT_1_R). Its production is elevated in chronic human HF [173]. Adrenal β-arrestin1-dependent AT_1_R signaling promotes aldosterone synthesis and secretion both in vitro and in vivo [174]. Moreover, inhibition of this pathway in the adrenal cortex in vivo markedly attenuates post-MI HF [175,176]. In fact, adrenal β-arrestin1 is absolutely essential for disease-associated hyperaldosteronism, since its absence results in normal circulating aldosterone levels even in the presence of MI [102]. Importantly, catecholamine-activated βARs were recently shown to enhance AT_1_R-dependent aldosterone production via GRK2-mediated receptor crosstalk in adrenocortical cells [177]. Thus, it appears that the catecholamine and aldosterone secretion pathways are intricately interposed in the adrenal gland via β-arrestin-dependent signaling [178,179].

The direct effects of aldosterone or of its receptor (MR) on cardiac AR signaling and function have not been well studied. Studies from our laboratory have revealed that aldosterone can impair the cardiac β_2_AR anti-fibrotic signaling mediated by cAMP via osteopontin transcriptional upregulation [148]. Osteopontin, a drug previously mentioned, is a pro-inflammatory/pro-fibrotic cytokine that has been shown to derange β_2_AR signaling in various tissues, such as bone cells. We recently showed that, in H9c2 cardiomyocytes, its CRISPR/Cas9-mediated genetic deletion leads to augmented cAMP signaling by β_2_AR, which protects against TGFβ-induced fibrosis via Epac1 activation [148]. In addition, osteopontin seems to facilitate GRK2-mediated desensitization of this receptor, i.e., β_2_AR-G_s_ protein uncoupling, which further diminishes the anti-fibrotic actions of β_2_AR in cardiomyocytes and cardiac fibroblasts [86].

**Figure 1:**
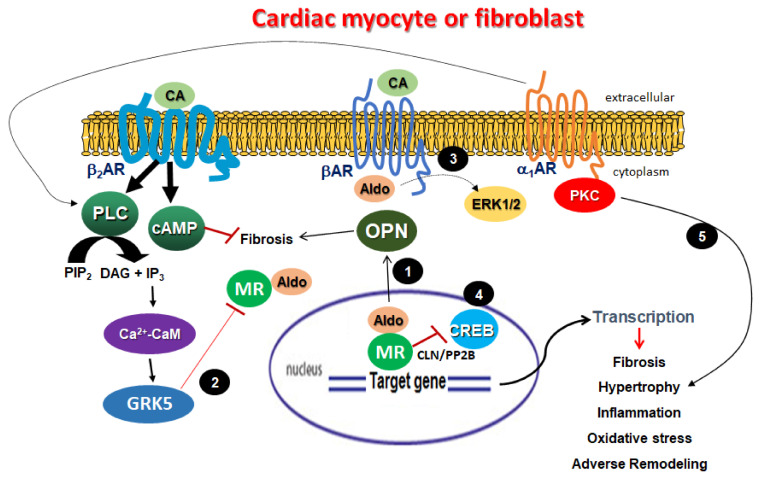
Five emerging molecular points of aldosterone–AR signaling crosstalk in the heart. 1. OPN is transcriptionally upregulated by aldosterone and, in turn, dampens the cAMP-mediated anti-fibrotic signaling of the β_2_AR; 2. β_2_AR-stimulated GRK5 “sheds” from the plasma membrane to the cytoplasm, wherein it phosphorylates and inhibits MR (prevents MR nuclear translocation); 3. Aldosterone enhances catecholamine (βAR)-mediated ERK activation resulting in enhanced fibrosis; 4. MR inhibits CREB (usually activated by βARs) via CLN/PP2B; 5. α_1_AR-activated PKC enhances aldosterone-induced hypertrophy and adverse remodeling. Adapted from Parker at al., 2018 [3].

Interestingly, there appears to be a regulatory feedback loop between β_2_AR and aldosterone in the myocardium. Not only does aldosterone inhibit β_2_AR signaling via upregulation of osteopontin, but the β_2_AR opposes aldosterone signaling in the heart via the cardiac MR by activating GRK5 [180] (Figure 1). Indeed, GRK2 phosphorylates and desensitizes the cardioprotective G protein-coupled estrogen receptor (GPER) but GRK5 phosphorylates the MR in the cytoplasm to induce MR cytosolic retention and blockade of its transcriptional activity (Figure 1). Of note, this so-called “non-canonical” effect of GRK5, given that the MR is not a GPCR, is antithetical to the kinase’s nuclear/genomic effects as a class II histone deacetylase (HDAC) kinase, which are largely harmful, as they promote pathological hypertrophy [181]. Indeed, subcellular localization of GRK5 seems to dictate the nature of the cardiac actions of this kinase: inside the nucleus it promotes maladaptive hypertrophy but, in the cytoplasm, it may afford cardio-protection against aldosterone [180,181,182,183]. It is important to note that the overall balance of GRK5’s effects on the myocardium, i.e., whether it is positive or negative, remains controversial with some studies reporting a beneficial, protective role for GRK5 in the myocardium, including attenuation of atherosclerosis and cardiac nuclear factor (NF)-κB NF inhibition [184,185,186,187], but some others reporting adverse remodeling effects like promotion of fibrosis [188,189]. Finally, regarding the precise signaling mechanism underlying the inhibitory phosphorylation of the MR by GRK5 in the cytoplasm, we know that β_2_AR needs to activate a non-conventional PLCβ-Ca^2+^-CaMKII pathway, normally activated by G_q_ protein-coupled receptors, which then triggers the “shedding” of GRK5 from the cell membrane, where it normally resides bound to membrane phospholipids via a polybasic amino acid region of its C-terminus, into the cytoplasm [180,190] (Figure 1). Interestingly, the β_1_AR seems incapable of activating this signaling pathway and, in fact, it may even inhibit it [180,191].

Beyond GRK5 and osteopontin, there are some other molecular/signaling “points” of crosstalk between aldosterone and ARs that may operate in myocardial cells. One of them is ERK phosphorylation and activation. Indeed, aldosterone antagonism with eplerenone has been shown to inhibit βAR-dependent cardiac fibrosis in primary cardiac fibroblasts, as well as in rat left ventricles via blockade of isoproterenol-induced ERK phosphorylation (Figure 1), of 11β-hydroxysteroid dehydrogenase type I (11β-HSD1), and of collagen expressions [192]. This suggests that aldosterone can mediate catecholamine-elicited pro-fibrotic signaling in the heart. cAMP-responsive element binding protein (CREB) phosphorylation and activation might be another point where aldosterone and βAR signaling intercalate at (Figure 1) [193]. CREB is a pivotal transcription factor that crucially mediates βAR-induced gene transcription/expression and MR seems capable of inhibiting CREB activity via calcineurin/protein phosphatase (PP)-2B activation [193]. Nevertheless, whether this occurs in cardiac cells or in the myocardium in vivo remains to be seen. Of note, GRK2 was recently shown to mediate sustained activation of α2AR-stimulated CREB in neuronal cells [194]. Furthermore, α_1_AR signaling may also crosstalk with aldosterone and the MR at the level of PKC activation (Figure 1). In fact, it has been reported that both α_1_AR activation by phenylephrine and PKC activation with phorbol esters upregulates corticosteroid pro-hypertrophic signaling in cardiomyocytes, while, in turn, aldosterone (and corticosterone) promotes phenylephrine-elicited hypertrophy and other remodeling effects in neonatal rat ventricular myocytes, in part thanks to hypertrophy-induced cardiac MR upregulation and serum/glucocorticoid-induced kinase (SGK)-1 activation [195].

## 5. Conclusions and Future Perspectives

More studies are warranted to further elucidate the molecules representing signaling connections between aldosterone–MR and AR signaling in the heart in different subcellular locations, including mitochondria. Given that the MR is not the only receptor activated by aldosterone, the picture can get quite complicated. Only a combination of in vitro and in vivo studies in both animal models and humans can help bring it into a better focus. Moreover, by studying the clinical effects of mineralocorticoid antagonists in HF patients, indirect insights into the role aldosterone plays in adrenergic regulation of cardiac function and morphology can be gleaned.

What is known with certainty is that the cardiac MR displays significant bidirectional signaling crosstalk with various GPCRs, including the ARs. In other words, aldosterone modulates AR function, number, and signaling, but also ARs modulate the actions of aldosterone (through MR) in the heart. Moreover, the effects of aldosterone in mitochondrial dysfunction have been proposed, even if the molecular mechanisms underlying these effects are not yet totally understood. The advent of novel, non-steroidal aldosterone antagonists (e.g., finerenone), which are purportedly more potent and specific inhibitors of the MR, represent another opportunity to gather novel insights into the interplay between AR and aldosterone signaling in the failing myocardium. Furthermore, the use of mitochondria as a pharmacological target will pave the road to new pharmacological therapies in this condition. Moreover, delineation of all signaling crosstalk mechanisms connecting aldosterone/MR and ARs in the heart has the enormous potential for delivering novel, safer, and more effective therapies for human chronic HF towards achieving the “holy grail” of precision medicine also for heart disease. Aided by methodological and technological advances in the field of GPCR structural biology, physiology, and pharmacology, future studies on the aldosterone–AR signaling reciprocity in different subcellular compartments, including mitochondria, might one day lead to the addition of better, more targeted, and personalized weapons into the future cardiologist’s therapeutic arsenal.

## Data Availability

Not applicable.

## Abbreviations

Please see text for details and for all other molecular acronym descriptions:

Aldo	Aldosterone
Ca^2+^-CaM	Calcium-bound calmodulin
CA	Catecholamine
cAMP	Cyclic 3′,5′-adenosine monophosphate
CLN	Calcineurin
CREB	cAMP response element-binding protein
DAG	2′-Diacylglycerol
ERK	Extracellular signal-regulate kinase
GRK5	G protein-coupled receptor kinase-5
IP_3_	Inositol 1′,4′,5′-trisphopshate
OPN	Osteopontin
PIP2	Phosphatidylinositol 4′,5′-bisphosphate
PKC	Protein kinase C
PLC	Phospholipase C
PP2B	Protein phosphatase-2B
